# Risk factor of withdrawal syndrome in the paediatric ICU

**DOI:** 10.1186/cc13611

**Published:** 2014-03-17

**Authors:** C Tanaka, N Shimizu, O Staito, M Motomura, I Watanabe

**Affiliations:** 1 Japanese Musashino Red Cross Hospital, Tokyo, Japan; 2 Tokyo Metropolitan Children's Centre, Tokyo, Japan

## Introduction

Critically ill paediatric patients supported on mechanical ventilation frequently receive analgesia and sedation. Iatrogenic withdrawal syndrome occurs with the abrupt discontinuation or too rapid weaning of opioids and benzodiazepines. We induce the Withdrawal Assessment Tool-1 (WAT-1) [1] to evaluate children during weaning from analgesics and sedatives. The patient is diagnosed with withdrawal syndrome when the score is 3 or >3. We compared the subjects who ever had a score over 3 and those with lower scores and assessed the risk factors and outcome of withdrawal syndrome between two groups.

## Methods

A total of 932 patients were admitted to our PICU from 1 October 2011 to 31 March 2013. Of these, 127 paediatric patients were supported on mechanical ventilation on the first day in the PICU and received intravenous analgesics and sedatives. A retrospective review of a prospectively collected database. The statistical method was the Mann-Whitney *U *test, and *P *< 0.05 was considered statistically significant.

## Results

Twenty-eight patients were scored with the WAT-1 during weaning from the drugs. Median age was 14 months (1 to 73 months) and the most common reasons for ventilation were airway trouble and pneumonia. Eight of 28 were diagnosed withdrawal syndrome, 20 were not. There were no significant differences in age, body weight, cumulative morphine and benzodiazepine dose before weaning. In the withdrawal group, the lactate level, catecholamine index, Paediatric Index of Mortality 2 and heart rates were greater when they were admitted to hospital. Further, there are longer length of PICU stay and shorter ventilator-free days for patients with WAT-1 score >3. But all of them are alive at 28 days, so there is no difference in 28-day mortality.

## Conclusion

Our study suggest that severely ill paediatric patients tended to suffer from withdrawal syndrome and then resulted in short ventilator-free days and longer PICU stay. But there is no difference in mortality.
